# An Ethnobotanical Study of Medicinal Plants in the Greek Islands of North Aegean Region

**DOI:** 10.3389/fphar.2018.00409

**Published:** 2018-05-23

**Authors:** Evangelos Axiotis, Maria Halabalaki, Leandros A. Skaltsounis

**Affiliations:** Department of Pharmacognosy and Natural Products Chemistry, Faculty of Pharmacy, National and Kapodistrian University of Athens, Athens, Greece

**Keywords:** ethnopharmacology, traditional medicine, Near East Greek islands, North Aegean Sea, ethnobotany

## Abstract

Greek islands of the North Aegean Region are a group of nine inhabited islands (Lemnos, Agios Efstratios, Lesvos, Chios, Psara, Oinousses, Samos, Ikaria, and Fourni) located in the northern part of the Aegean Sea, close to Asia Minor. Each island of this region can be considered autonomous in terms of culture and biodiversity. With this work we try to evaluate the status of the traditional uses of medicinal plants in this region. Endemic and endangered species such as *Sideritis sipylea* Boiss., *Origanum sipyleum* L., *Thymus sipyleus* Boiss., *Pistacia lentiscus* L., *Verbascum ikaricum* Murb., are still used by locals to treat different ailments. Moreover, the use of some species for the treatment of specific diseases has been reported for the first time. We report about 109 wild plants of medicinal importance, from 52 families, listing their uses for therapeutic purposes and galenic preparations provided by local medical doctors and pharmacists. The information we include was derived from literature sources and additionally collected through semi-structured interviews conducted on 200 informants (100 men and 100 women). Additionally, informant consensus factor (FIC) and UV value were calculated for the medicinal plants in the current study in relation with the diseases treated. This research confirms the importance of the medicinal plants and the diffusion of their use in traditional medicine within this region. This ethnopharmacological survey is a fundamental step for the preservation of the local knowledge both for further scientific research and for the protection of endangered and endemic medicinal plants.

## Introduction

Greece holds a unique position with respect to the number of plant species and subspecies compared to other areas of the globe. This depends on the variety of habitats, as well as on geological history, climate conditions, and geographical position within the Mediterranean. Thus, it is noted for its high plant species diversity (5800 species and 1893 subspecies) and endemism (22.2% of all species present with 1278 species and 452 subspecies) (Davis, 1965–1986; Tutin et al., 1968–1980; Strid, 1986; Strid and Tan, 1991, 1997, 2002; Georgiou and Delipetrou, 2010).

The Northeast Aegean islands offer a unique ecosystem with significant “hotspots” for various plant diversification responses and endemism. This depends on the fact that Aegean Sea is an archipelago of continental islands placed on the conjunction of Europe, Asia, and Africa (Gogou et al., 2016). The available “ecological space” with “environmental heterogeneity” in addition with the “land-bridge system” with the continents determined a high floristic diversity and endemism (Kallimanis et al., 2011). Several botanical studies have been conducted on these islands (Christodoulakis, 1986, 1996; Panitsa et al., 1994, 2006; Panitsa and Tzanoudakis, 1998, 2001; Snogerup et al., 2001; Bazos, 2005). However, there is limited information regarding the medicinal plants and traditional plant remedies in this area.

Nowadays the use of plants and traditional medicine plays an important role for the discovery of new pharmacological agents. Ethnopharmacology represents a multidimensional approach, shaped by tradition and science that can improve our knowledge of plant use and local meaning of health and disease (World Health Organization [WHO], 2002). The aim of this study is threefold: to present a complete list of the medicinal plants used in traditional medicine in the islands of Northeast Aegean Sea, preserve information about their use and, lastly, highlight the use in traditional medicine of endemic and endangered plant species, in order to prevent their extinction.

## Materials and Methods

### Study Area

**FIGURE 1:**
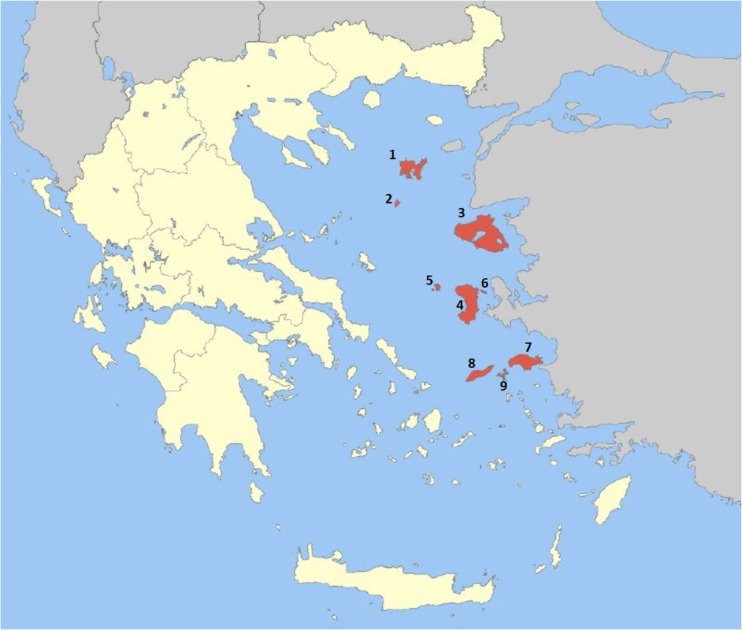
Geographical location of the study area (Islands in red). Collection sites of the information are as follows. (1) Lemnos, (2) Agios Efstratios, (3) Lesvos, (4) Chios, (5) Psara, (6) Oinousses, (7) Samos, (8) Ikaria, (9) Fourni (http://www.pvaigaiou.gov.gr).

The current study was conducted on the islands and islets of the northeast edge of the Aegean Sea and is dominated by the islands of Lemnos, Agios Efstratios, Lesvos, Chios, Psara, Oinousses, Samos, Ikaria, and Fournoi (**Figure 1**). The geographical coordinates of the islands are represented in Supplementary Table 4. The region covers an area of 3835 km^2^ and has approximately 2500 vascular plant species.

The terrain of the islands is 33% mountainous, 35% hilly, and 32% flat. The surface area and the maximum elevation ranged from 40 km^2^ for Psara and Fourni to 1633 km^2^ for Lesvos and from 0 to 1433 m of height (Kerketea mt, Samos), respectively (Higgins and Higgins, 1996). The islands can be grouped in two phytogeographical zones among the 13 existing in Greece (Supplementary Figure 1); the zone of the Northern Aegean **(NAe)** with Lemnos and Agios Efstratios and the zone of the Eastern Aegean **(EAe)** with Lesvos, Psara, Chios, Oinousses, Samos, Ikaria and Fournoi, with specific climatic differences (Rauh, 1949). The palaeography and the geological events that separated these islands from Anatolian mainland, certain environmental parameters (temperature and humidity) and habitat diversity in well-defined fragmented areas, are among the causes of the high endemism, floral richness and diversification in quantity and quality of many secondary metabolites that characterize the pharmacological properties of many species of the study area (Panitsa et al., 2010).

### Methodology

The information summarized in the present paper was compiled from 15 randomly selected villages from the islands of the surveyed area (**Figure 1**). The methodology followed in the field surveys is based on Fujita et al. (1995). The data was collected through semi-structured interviews performed with local people (Martin, 1995). After explaining the purpose of our research, a questionnaire in Greek was administered to the informants including questions about their age, education, and interest in traditional medicine. A total of 200 people was surveyed, 100 women and 100 men, with an average age of 40. The informants with knowledge about medicinal plants were questioned multiple times, and, during the interviews, local names of the plants, utilized parts, preparation methods and traditional cultivation techniques were recorded. The majority of these skillful informants were medical doctors, pharmacists, and farmers. All the results are summarized in Supplementary Table 1. The data acquired for each plant includes family, botanical name, local name in Greek, locality, voucher number, parts used and their preparation, therapeutic effect and ailments treated. The plant families were listed in alphabetical order.

Field trips were conducted with the interviewees to collect specimens of the plants (with the exception of the endangered ones). A photographic archive of the observed species was created to help the identification. The specimens were collected and herborized by the department’s herbarium with a specific voucher number. Furthermore, we examined the therapeutic effect and preparations of each plant based on the informants’ feedback and on literature records.

For the analysis of the use of the medicinal plants against specific diseases, we used informant consensus factor (FIC), summarized in Supplementary Table 3 (Trotter and Logan, 1986; Heinrich, 2000). Moreover, the Use Value (UV) was calculated to demonstrate the relative importance of the species known locally, summarized in Supplementary Table 1. The plant species scientific names were verified according to the Plant List and to the International Plant Name Index. Information that was not possible to confirm was not recorded. Moreover, in Supplementary Table 1, we reported some of the most important bibliographic references for plant species and ailments treated that were not mentioned by the informants (Snogerup and Snogerup, 1987, 1993; Panitsa et al., 1994, 2006, 2010; Snogerup et al., 2001; Bergmeier et al., 2003; Bazos, 2005; Bourbonnais-Spear et al., 2006; Saliaris, 2008; Axiotis and Axiotis, 2012; Strid, 2016).

## Results and Discussion

In the context of the current survey numerous medicinal plants were ethnopharmacologicaly investigated (in Supplementary Table 1). The interviews indicated that 109 wild plant species from 52 different families are being used for medicinal purposes. Lamiaceae family is represented by the highest number of species (17), such as the endemic and endangered *Sideritis sipylea* Boiss. Asteraceae family is represented by 12 species. Brassicaceae and Rosaceae by 5 species. The rest of the plant families are shown in Supplementary Figure 2. Most of the plant parts were used to treat different diseases and they were mostly stored in glass bottles as homemade dry powders obtained by crushing down well-dried plant materials; the most frequently used parts are leaves (22,8%), roots (12,78%), flowers (11,41%), essential oils (7,30%), fruits (6,84%), and barks (5%). The most common preparation is the decoction.

Interestingly, *Hypericum perforatum* L. and *Verbascum ikaricum* Murb. are kept in olive oil and used as solutions for wounds and sun burns. Pharmacists from Lesvos, Chios and Samos, still use these preparations as traditional therapy for skin wound healing in combination with modern treatments. Furthermore, clinicians from Lesvos use the powder of the root of *Alkanna tinctoria* for skin regeneration after injuries. They observed a combined antibacterial and antipruritic action, which reduces the healing time of the wound. It is important to note that *Sideritis sipylea* Boiss., *Origanum sipyleum* L., *Thymus sipyleus* Boiss., which are endemic and endangered plants are mostly used by the local people of Lesvos, Chios, and Ikaria with many curative purposes, especially for the infections of the respiratory and gastrointestinal tract as indicated from the UV values (Supplementary Table 1).

Furthermore, the resin of *Pistacia lentiscus* L., unique product globally of the Chios island is widely used against diarrhea, as expectorant and for ulcer healing. The use of the water extract of the roots of *Anthyllis hermanniae* L. as an efficient natural remedy against kidney stones in Lesvos and Lemnos, is reported here for the first time. It was also observed that local people, especially farmers, use the same name for different plant species. For example, *Cistus creticus* L. and *Cistus salvifolius* L. are both called “Aksìstaros”; *Origanum vulgare* L. and the endemic and endangered *Origanum sipyleum* L. are both called “Rìgani.” This lack of distinction by the local population exacerbates the risks for the endangered species.

**FIGURE 2:**
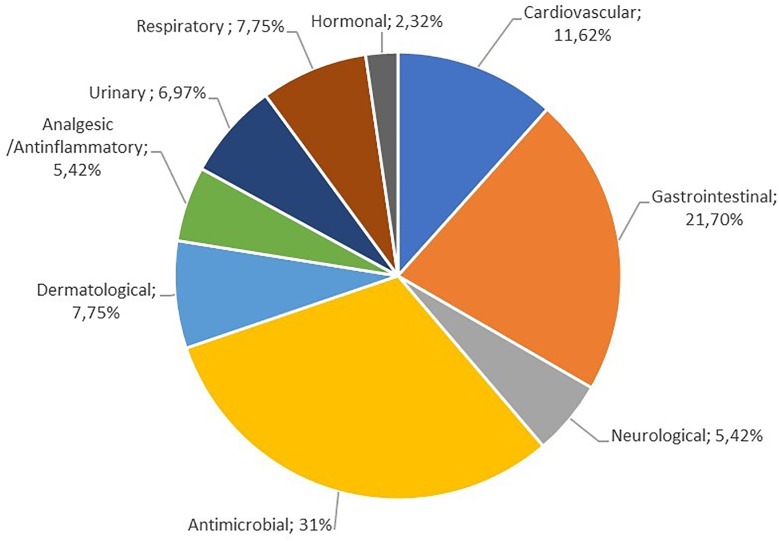
Distribution of ailments treated versus plant species used.

Very interesting is the distribution of ailments treated versus plant species used. As it is presented in **Figure 2**, locals are choosing herbal remedies mainly for antimicrobial activity, gastrointestinal disorders, respiratory, inflammatory, cardiovascular diseases, and skin burns. In Supplementary Table 3, FIC values of categories of ailments are summarized. Hormonal diseases have the highest FIC value (0,722), while the lowest belongs to the cardiovascular diseases (0,161). Moreover, among the species recorded (Supplementary Table 1), the highest UV were calculated for *Matricaria chamomilla* L. (0.75) and *Lavandula stoechas* L. (0.64). From the endemic species the highest UV is recorded for *Sideritis sipylea* Boiss. (0.31).

Essential oils are also common in the region, mainly derived from aromatic plants of Lamiaceae family and they are produced by water or steam distillation. The oils are usually used as drops directly on the skin or in galenic formulations, for skin infections or burns. In some cases, they are used for stomach pain (*Anthemis* sp.) or even for urinary infections (*Sideritis sipylea* Boiss.). The most common plants used for producing essential oils are *Salvia fruticosa* Miller, *Salvia officinalis* L., *Origanum vulgare* L., *Sideritis sipylea* Boiss., and *Lavandula stoechas* L. Furthermore, it has been reported a traditional method for the production of rose oil of the flower petals of *Rosa damascena* Mill. in a small village of Lesvos. The oil is used for external burns or skin inflammations.

The pharmacological properties of medicinal plants presented in the current study has been also verified by published data, in several cases. For example, the essential oil of *Laurus nobilis* L., used as anti-inflammatory is reported by Sayyah et al. (2003). The expectorant effect of *Cistus creticus* L. and the use of *Mentha spicata* L. in cases of common cold have been reported by Fakir et al. (2009). Tonic effects of *Rosmarinus officinali*s L. and the diuretic effects of *Raphanus raphanistrum* L. have been reported by Bruni et al. (1997). The essential oil of *Sideritis* species presents an important antimicrobial and anti-inflammatory activity and it has been previously reported by Aligiannis et al. (2001).

Generally, these plants are distributed mainly in Lesvos, Chios, Samos, and Ikaria and the majority are wild. A serious threat for some of these plants, is that they are collected in large amounts for the local markets; some of them, such as *Sideritis sipylea* Boiss.*, Origanum sipyleum* L.*, Thymus sipyleus* Boiss., are in fact listed in the book of the Red List of Threatened Species. Many species, such as *Origanum vulgare* L. and *Paeonia mascula* (L.) Mill. subsp. *mascula* are not yet listed but their population is in a critical condition. Endemic medicinal plants of the research area are also annotated in Supplementary Table 2.

A very important issue is the increased use of medicinal plants in these islands which has led to heavy pressure on the native populations of many species. The local population must acknowledge their endangerment and should cultivate them, in order to protect their populations from extinction. *Sideritis sipylea* Boiss. is one of the over harvested species. Habitat loss and deforestation in Lesvos, Chios, and Samos resulted in a general degradation of the ecosystems that could lead to the extinction of many species.

In conclusion, from this brief report is clear that medicinal plants are an important element of indigenous medical system of Northeast Aegean islands. In spite of the fact that the local population has access to modern medicines, many people continue to depend, at least for the treatment of some diseases, on herbal remedies. These therapies represent for many local doctors a low-cost alternative. The population of many endemic species such as *Sideritis sipylea* Boiss.*, Origanum sipyleum* L., are in a critical condition due to unsustainable harvesting techniques, habitat destruction, and absence of cultivation. The endemic species of the area have medicinal properties dependent on the metabolites which respond to environmental stimuli that may be absent under culture conditions. Because of this it is very important to educate local people about respecting their natural habitats and strengthen the legislation against the illegal trade of endemic and endangered plants in the local and national market.

## Author Contributions

EA contributed to the study conception and design of the methodology. Moreover, he was responsible for the analysis and the interpretation of data. MH contributed substantially to supervising the work and drafting the manuscript. LS contributed to the critical and final revision of the manuscript.

## Conflict of Interest Statement

The authors declare that the research was conducted in the absence of any commercial or financial relationships that could be construed as a potential conflict of interest.
