# Supplementary material for: An Ethnobotanical Study of Medicinal Plants in the Greek Islands of North Aegean Region
Source: Front Pharmacol. 2018 May 23;9:409. doi: 10.3389/fphar.2018.00409 (PMC5974156; doi:10.3389/fphar.2018.00409)

***Supplementary Material***

**An Ethnobotanical Study of Medicinal Plants in the Greek Islands of North Aegean Region**

**Evangelos Axiotis, Maria Halabalaki^*^ and Leandros A. Skaltsounis**

***Correspondence:** Maria Halabalaki, mariahal@pharm.uoa.gr

**Table S1.** Medicinal plants of research area

| **Plant Family** | **Botanical name (Voucher specimen)** | **Local names^a^**  **(number of the localities referring to the map)^b^** | **Plant part used^c^** | **UV***  **(Use Value)** | **Ailments treated, therapeutic effect and preparations based on ethnopharmacological survey** | **Related literature** |
| --- | --- | --- | --- | --- | --- | --- |
| Alliaceae | *Allium ampeloprasum* L.  **(KL183)** | Αγριόπρασο  (Agriòpraso)  **(3, 4, 5, 7, 8)** | AeP | 0.24 | Decoction of the whole plant as fungicide, antiseptic (candidiasis, vaginitis), anthelmintic | Khan and Sharma, 2016 |
| Amaranthaceae | *Amaranthus retroflexus* L.  **(AXL035)** | Βλίτο  (Vlìto)  **(1, 3, 4, 7, 8)** | AeP, Lf | 0.07 | All plant as hemostatic (poultice). As decoction for diarrhoea |  |
| Anacardiaceae | *Pistacia lentiscus* L.  **(KL191)** | Σχίνος  (Schìnos)  **(3, 4, 6, 7, 8, 9)** | Lf, Rn | 0.40 | Leaves for hair growth. Resin against diarrhoea, as expectorant, diuretic and for ulcer healing | E.M.A^*^ |

| Anacardiaceae | *Rhus coriaria* L.  **(AXL037)** | Σουμάκι  (Sumàki)  **(3, 7)** | Sd, Lf | 0.33 | Decoction of leaves and fruits as diuretic and against dysentery. As ointment against conjunctivitis |  |
| --- | --- | --- | --- | --- | --- | --- |
| Apiaceae | *Foeniculum vulgare* Miller  **(AXL001)** | Μάραθο  (Màratho)  **(1, 2, 3, 4, 7, 8, 9)** | Lf, Fr | 0.14 | Fruits for aerophagy. Decoction from leaves as diuretic (2 teaspoons of plant powder in 1/2 lt of water). Essential oil as expectorant in cough associated with common cold | E.M.A^*^ |
| Apiaceae | *Scandix pecten-veneris* L.  **(F019)** | Χάτζικας  (Chàtzikas)  **(1, 2, 3, 4, 5, 6, 7, 8, 9)** | AeP | 0.04 | The fresh aerial parts are cooked and eaten for antiseptic and antimicrobial activity |  |
| Apiaceae | *Tordylium apulum* L.  **(AXL031)** | Καυκαλήθρα  (Kaukalìthra)  **(1, 2, 3, 4, 5, 6, 7, 8, 9)** | AeP | 0.08 | The aerial parts are boiled as antiseptic and antimicrobial of gastrointestinal tract |  |
| Apocynaceae | *Vinca major* L.  **(AXL038)** | Βίνκα η μείζων  (Vìnca)  **(1, 2, 4, 7, 8)** | Lf | 0.14 | Dry leaves as poultice for haemorrhages and insect bites and after boiling for dermatitis eczemas, acne |  |
| Asparagaceae | *Asparagus acutifolius* L. **(NEK029)** | Σπαράγγι  (Sparàgki)  **(1, 2, 3, 4, 5, 6, 7, 8, 9)** | Rt | 0.22 | The roots are cooked and eaten as diuretic.and as tincture for kidney diseases and cardiotonic |  |
| Asphodelaceae | *Asphodelus aestivus* Brot.  **(UoA003)** | Ασπούρδουλας  (Aspùrdulas)  **(1, 3, 4, 7, 8)** | Rt | 0.35 | Roots as powder with boiled water as antispasmodic and diuretic. Freshly cut roots for psoriasis, skin wounds (a mix with masticha of Chios, olive oil, bees wax and incense) as ointment |  |
| Asteraceae | *Anthemis rosea* Sm subsp. *rosea*  **(AXL039)** | Ανθέμιδα η ρόδινη (Anthèmida)  **(7)** | Fl | 0.01 | The essential oil for stomach pain, nervous ailments |  |

| Asteraceae | *Artemisia arborescens* L.  **(KL202)** | Αψιθιά  (Apsithià)  **(3, 4, 7, 8)** | AeP | 0.12 | Essential oil for skin burns, microbial dermatitis, sun burns |  |
| --- | --- | --- | --- | --- | --- | --- |
| Asteraceae | *Calendula arvensis* L.  **(AXL041)** | Αγροδίαιτος  (Agrodìetos)  **(1, 2, 3, 4, 5, 6, 7, 8, 9)** | AeP | 0.18 | All plant and especially tea from flowers (dry or fresh) for wounds, hemorrhoids, antispasmodic |  |
| Asteraceae | *Carthamus lanatus* L.  **(AXL013)** | Σταυράγκαθο  (Stayràgkatho)  **(3, 4, 7, 8)** | Lf | 0.13 | 10-12 leaves of the plant grinded and 1 teaspoon boiled, filtered, for wounds as analgesic and anti-inflammatory |  |
| Asteraceae | *Centaurea cyanus* L. **(AXL042)** | Κενταυρία η κυανή  (Kentaurìa)  **(3, 7)** | Sd, Fl | 0.21 | Seeds boiled as laxative for kids. Flowers in distilled water for conjunctivitis as eye patch |  |
| Asteraceae | *Cichorium spinosum* L.  **(KL011)** | Σταμναγκάθι της Κρήτης (Stamnagkàthi)  **(5, 4, 7, 8, 9)** | Rt, Lf, Sh | 0.11 | Leaves boiled for skin inflammations and oedemas (5ml of boiled leaves in 250ml of water) |  |
| Asteraceae | *Eupatorium cannabinum* L. **(AXL040)** | Ευπατόριο το καννάβινο (Eupatòrio)  **(3, 7)** | AeP, Rt | 0.19 | Tea from leaves for fever, common cold and flu. Powder of roots as tonic |  |
| Asteraceae | *Helichrysum stoechas* DC. **(AXL015)** | Αμάραντο  (Amàranto)  **(1, 3, 4, 5 ,6, 7, 8, 9)** | Fl (e/o) | 0.55 | Flower boiled in water for common cold and expectorant (decoction). Essential oil as antimicrobial for skin wounds |  |
| Asteraceae | *Matricaria chamomilla* L. **(AXL023)** | Χαμομήλι  (Chamomìli)  **(1, 2, 3, 4, 5, 6, 7, 8, 9)** | Fl (e/o) | 0.75 | Tea from flowers and essential oil as tranquilizer, antiseptic, antispasmodic, vasodilator, anti-inflammatory | Srivastata et al., 2010; Strid, 1986; E.M.A^*^ |

| Asteraceae | *Silybum marianum* (L.) Gaertn.  **(AXL016)** | Αγκαθούρα  (Agkathùra)  **(1, 2, 3, 4, 7)** | AeP | 0.23 | All plant boiled in water for liver diseases (hepatitis, liver cirrhosis) Tea preparation for symptomatic relief of digestive disorders with a sensation of fullness, bloating and flatulence | Bahmani et al., 2015; E.M.A^*^ |
| --- | --- | --- | --- | --- | --- | --- |
| Asteraceae | *Taraxacum hellenicum* Dahlst.  B hj  **(AXL043)** | Πικροράδικο  (Pikroràdiko)  **(1, 2, 3, 4, 5, 7)** | Lf, Rt | 0.10 | Tea of the plant as laxative and for chronic skin diseases |  |
| Asteraceae | *Urospermum picroides* (L.) Scop. ex F.W.  **(AXL025)** | Άγριος ζόχος  (Zohòs)  **(1, 2, 3, 4, 5, 6, 7, 8, 9)** | AeP | 0.16 | Cooked and eaten for antifungal activity. Externally as antiseptic |  |
| Boraginaceae | *Alkanna tinctoria* Tausch  **(AXL034)** | Αλκάννα  (Alkànna)  **(1, 3, 4, 5, 7, 8, 9)** | Rt | 0.34 | Powder of root externally for hemorrhoids, chronic ulcers, antibacterial and antipruritic action |  |
| Brassicaceae | *Alliaria officinalis* Andrz. ex DC.  **(AXL044)** | Αλλιάρια  (Alliària)  **(3, 4, 7)** | Lf, Sh, Rt | 0.41 | Leaves and shoots are boiled (poultice) for eczemas. Roots with hot olive oil for bronchitis (poultice) |  |
| Brassicaceae | *Capsella bursa-pastoris* (L.) Medik.  **(KL084)** | Ταγάρι του βοσκού  (Tagàri)  **(1, 2, 3, 4, 5, 6, 7, 8, 9)** | AeP, Fr | 0.15 | All plant as laxative and hemostatic (decoction). Dry plant decoction for menorrhagia, rhinorrhagias, cystitis, urolithiasis (non-more than 1 year) | E.M.A^*^ |
| Brassicaceae | *Nasturtium officinale* R.Br.  **(AXL046)** | Νεροκάρδαμο  (Nerokàrdamo)  **(1, 2, 4, 7, 8)** | AeP, Lf, Fr | 0.16 | Plant rich in vitamin C. Juice from leaves for kidney and lung diseases |  |
| Brassicaceae | *Raphanus raphanistrum* L. **(AXL047)** | Ρεπάνι  (Repàni)  **(1, 3, 4, 7, 8)** | AeP | 0.25 | Aerial parts are cooked as diuretic and antiscorbutic | Fakir et al., 2009 |

| Brassicaceae | *Sisymbrium officinale* (L.) Scop.  **(AXL048)** | Σισύμβριο  (Sisìmvrio)  **(1, 2, 3, 4, 5, 6, 7, 8, 9)** | AeP, Sh | 0.21 | Water extract as diuretic and expectorant (for the relief of throat irritation such as hoarseness and dry cough) | E.M.A^*^ |
| --- | --- | --- | --- | --- | --- | --- |
| Capparaceae | *Capparis spinosa* L.var.*spinosa*  **(AXL021)** | Κάππαρις  (Càpparis)  **(1, 3, 4, 5, 7, 8, 9)** | Rt, Fl, Lf | 0.41 | Decoction of root barks as analgesic, anthelmintic, diuretic and vasoconstrictor. Pickles of the flowers for stomach pain |  |
| Caprifoliaceae | *Sambucus nigra* L.  **(Ath011)** | Σαμπούκος ο μελανός (Sambùkus)  **(1, 3, 7, 8)** | Fl, Sd | 0.23 | Flowers and fruits boiled (3-5g of dry parts in 150ml of water for 15min) for bronchitis, cough, fever and expectorants | E.M.A^*^ |
| Caryophyllaceae | *Cerastium glomeratum* Thuill. **(AXL049)** | Κεράστιο  (Keràstio)  **(1, 2, 3, 4, 5, 6, 7, 8)** | AeP | 0.09 | All plant juice for headache. As patch and as drops for nosebleed |  |
| Caryophyllaceae | *Herniaria hirsuta* L.  **(AXL050)** | Χερνιάρια  (Herniària)  **(1, 2, 3, 4, 5, 7, 8)** | AeP | 0.07 | The juice of the aerial parts as diuretic |  |
| Caryophyllaceae | *Saponaria officinalis* L.  **(Ath010)** | Σαπωναρία / Τσουένι  (Tsuèni)  **(3, 7)** | Rt, Lf | 0.12 | All plant boiled and used as expectorant |  |
| Caryophyllaceae | *Stellaria media* (L.) Vill.  **(AXL051)** | Στελλάρια  (Stellària)  **(1, 3, 4, 7, 8)** | AeP | 0.20 | All plant for every type of itch and as poultice for psoriasis, wounds |  |
| Chenopodiaceae | *Chenopodium album* L.  **(AXL052)** | Χηνοπόδιον  (Chinopòdion)  **(1, 3, 4, 7, 8, 9)** | Lf, Fr, Rt | 0.18 | Leaves and fruits as anthelmintic, antirheumatic, and teeth pain. Also, as a poultice for sun burns and oedemas. The juice of the roots is used for bloody diarrhoea | Lone et al., 2017 |
| Cistaceae | *Cistus creticus* L.  **(KL057)** | Aξίσταρος  (Aksìstaros)  **(1, 3, 4, 5, 6, 7, 8, 9)** | Lf, Bk | 0.53 | Leaves as poultice, as antiseptic and the tea as expectorant | Heinrich, 2000 |

| Cupressaceae | *Cupressus sempervirens* L. **(KL165)** | Κυπαρίσσι  (Kiparìsi)  **(1, 3, 4, 7)** | Cn | 0.18 | As decoction for common cold, diarrhoea, ulcer, constipation, enuresis nocturna, hemorrhoids | Tapondjou et al., 2005 |
| --- | --- | --- | --- | --- | --- | --- |
| Cupressaceae | *Juniperus oxycedrus* L.  **(Pang018)** | Κέδρος  (Kèdros)  **(3, 4, 7)** | Fr (e/o) | 0.29 | 30-60g of fruits in 1 lt of white wine for cystitis. Tincture of fruits for skin diseases and alopecia |  |
| Cyperaceae | *Cyperus rotundus* L.  **(AXL054)** | Ζιζάνιο  (Zizànio)  **(1, 2, 3, 4, 7, 8)** | Rt, Tb, e/o | 0.41 | The roots boiled in water as analgesic, antispasmodic. The essential oil as antitussive, diuretic and laxative |  |
| Datiscaceae | *Datisca cannabina* L.  **(AXL055)** | Δατίσκη η καννάβινος  (Datìski)  **(3, 7)** | Lf, Rt, Sh | 0.32 | Leaves for rheumatic pains, and teeth pain (poultice) |  |
| Dioscoreaceae | *Tamus communis* L.  **(KL051)** | Σβυρνιά  (Svirnià)  **(1, 2, 3, 4, 7, 8)** | AeP, Rt | 0.41 | The root in oil (tincture) for sun burns, contusions |  |
| Ephedraceae | *Ephedra foeminea* Forssk.  **(AXL060)** | Εφέδρα η γκριζωπή  (Efèdra)  **(1, 2, 3, 4, 5, 6, 7, 8, 9)** | Sh | 0.03 | Plant water extract for asthma, allergic rhinitis, allergic fever |  |
| Fabaceae | *Medicago sativa* L.  **(AXL061)** | Μηδική  (Midikì)  **(3, 4, 7, 8)** | Lf, AeP | 0.04 | Leaves as antianemic, anti-hemorrhagic (decoction). The whole plant cooked for menstruation problems | Mirzaei et al., 2015 |
| Fabaceae | *Melilotus indicus* (L.) All.  **(AXL062)** | Μελίλοτος  (Melìlotos)  **(1, 2, 3, 4, 7, 8)** | AeP, Gr | 0.10 | Grains in porridge for diarrhoea and stomach pain. A decoction of the aerial parts as laxative |  |
| Fagaceae | *Castanea sativa* Mill.  **(AXL063)** | Καστανιά  (Kastanià)  **(1, 3, 4, 7, 8)** | Lf, Fr,Bk | 0.31 | Leaves and fruits against diarrhoea (boiling for 2mins in 3 o 4 doses). Decoction as expectorant. Bark as antipyretic (decoction) |  |

| Fagaceae | *Quercus ilex* L.  **(AXL064)** | Δρυς  (Drìs)  **(1, 7, 8)** | Bk | 0.21 | Decoction for chronic diarrhoea and dysentery. For hemorrhoids (cataplasm) |  |
| --- | --- | --- | --- | --- | --- | --- |
| Fumariaceae | *Fumaria officinalis* L.  **(AXL065)** | Καπνιά  (Kapnià)  **(1, 2, 4, 7, 8)** | AeP | 0.03 | All plant for the treatment of obstructive liver (decoction) | Hentschel et al., 1995; E.M.A^*^ |
| Gentianaceae | *Centaurium erythraea* Rafn  **(AXL066)** | Θερμόχορτο  (Thermòchorto)  **(1, 3, 7, 8)** | AeP, Fl, Lf | 0.13 | All plant boiled for gastrointestinal and urinary tract (decoction). Externally fresh plant is used for wounds and ulcers as cataplasm | E.M.A^*^ |
| Geraniaceae | *Erodium cicutarium* (L.) L’Hér  **(AXL067)** | Ερόδιο  (Eròdio)  **(1, 2, 3, 4, 5, 6, 7, 8, 9)** | AeP, Lf, Rt | 0.14 | Infusion of the aerial parts for animal bytes and skin infections. Leaves in boiled water as antirheumatics |  |
| Geraniaceae | *Geranium dissectum* L.  **(AXL068)** | Γεράνιο  (Gerànio)  **(1, 2, 3, 4, 5, 6, 7, 8)** | AeP, Rt | 0.11 | Infusion of the aerial parts and the roots for diarrhoea, dysentery, gastroenteritis |  |
| Hypericaceae | *Hypericum perforatum* L.  **(Eb005)** | Αγούδουρας / βάλσαμο-(Agùduras)  **(1, 2, 3, 4, 6, 7, 8)** | AeP, Fl | 0.49 | Flowers in oil (balsam). Internally for duodenal ulcers and externally for wounds, sun burns, ulcers | Cayci and Dayioglu, 2009; Gambarana et al., 2001; Woelk et al., 1994; E.M.A^*^ |
| Iridaceae | *Crocus cartwrightianus* Herb.  **(AXL069)** | Κρόκος παλάσιι  (Kròkos)  **(8)** | An | 0.03 | All plant as analgesic (tea), antispasmodic, expectorant and tranquilizer (not more than 10g) |  |
| Lamiaceae | *Lavandula stoechas* L. subsp. *stoechas*  **(KL099)** | Λεβάντα ή Αβαγιανός – (Avagianòs)  **(3, 4, 6, 7, 8, 9)** | AeP, Lf, Fl | 0.64 | The essential oil from flowers has antiasthmatic, antiseptic and expectorant action. An infusion from flowers and branches for stomach ache, headache, menstrual regulation | Dadalioglu and Evrendilek, 2004 |
| Lamiaceae | *Melissa officinalis* L.  **(AXL002)** | Μελισσόχορτο  (Melisòchorto)  **(1, 3, 4, 7, 8)** | Fl, Lf | 0.58 | Boiled leaves and barks as antispasmodic and also as decoction for common colds. Herbal tea for relief of mild symptoms of mental stress and gastrointestinal complaints | Aubert et al., 2016; Koksal et al., 2011; E.M.A^*^ |

| Lamiaceae | *Mentha longifolia* (L.) Huds.  **(KL055)** | Μέντα  (Mènta)  **(1, 3, 7, 8, 9)** | AeP, Fl | 0.11 | Tea is used for headache, respiratory infections, dyspepsia, intestinal parasites |  |
| --- | --- | --- | --- | --- | --- | --- |
| Lamiaceae | *Mentha pulegium* L.  **(AXL003)** | Φλισκούνι  (Fliskùni)  (1, 3, 4, 5, 7, 8) | AeP, Fl | 0.64 | Tea is used for stomach pain and as an appetizer |  |
| Lamiaceae | *Mentha spicata* L.  **(KL054)** | Μέντα  (Mènta)  **(1, 3, 4, 7, 8)** | AeP, Fl | 0.32 | Tea is used for common cold and flu and essential oil as antifungal | Heinrich, 2000; Singh et al., 1994 |
| Lamiaceae | *Micromeria juliana* (L.) Benth. Ex Rchb.  **(AXL009)** | Πολύκομπο  (Polìkompo)  **(3, 4, 6, 7, 8)** | AeP | 0.03 | Decoction against warts (3times/die). The essential oil has strong antibacterial action |  |
| Lamiaceae | *Ocimum basilicum* L.  **(AXL070)** | Βασιλικός  (Vasilikòs)  **(1, 2, 3, 4, 5, 6, 7, 8)** | Fl, Bk | 0.28 | Infusion from flowers and branches for common cold and flu |  |
| Lamiaceae | *Origanum calcaratum* Juss.  **(AXL071)** | Ορίγανo το πληκτροφόρο-(Rìgani)  **(8)** | e/o | 0.12 | All plant for common cold, dyspepsia, dysmenorrhea (decoction) | Friedman, 2014 |
| Lamiaceae | *Origanum sipyleum* L.  **(CHI001)** | Ορίγανο η Σίπυλος  (Rìgani)  **(4, 7)** | e/o | 0.09 | All plant for common cold, dyspepsia, dysmenorrhea (decoction) | Friedman, 2014 |
| Lamiaceae | *Origanum vulgare* L.  **(Pang004)** | Oρίγανο το κοινόν  (Rìgani)  **(1, 3, 4, 7, 8)** | e/o, AeP | 0.31 | All plant for common cold, dyspepsia, dysmenorrhea (decoction) | Friedman, 2014 |
| Lamiaceae | *Rosmarinus officinalis* L.  **(KL163)** | Δενδρολίβανο  (Dentrolìvano)  **(1, 3, 4, 7, 8, 9)** | AeP, Fl (e/o), Lf | 0.42 | All plant boiled vapours for common cold and headache. Tea as antiseptic, antispasmodic, emmenagogue and tonic of nervous system. As adjuvant in the relief of minor muscle and articular pain | Fakir et al., 2009; E.M.A^*^ |
| Lamiaceae | *Salvia fruticosa* Mill.  **(KL053)** | Φασκόμηλο  (Faskòmilo)  **(1, 2, 4, 7, 8, 9)** | Lf(e/o) | 0.39 | Tea as tranquilizer, antipyretic, antiseptic and vasodilator. Essential oil is used for stomach pain, dysentery and anorexia. 100g of leaves in 1 lt of water for fungal infection of feet | Longaray et al., 2007; E.M.A^*^ |
| Lamiaceae | *Satureja thymbra* L.  **(AXL008)** | Σατουρεία η θύμπρα  (Saturìa)  **(3, 4, 5, 7, 8, 9)** | AeP | 0.25 | Boiled leaves for antibacterial action of gastrointestinal tract and expectorant. The essential oil has antifungal action |  |
| Lamiaceae | *Sideritis sipylea* Boiss.  **(NEK027)** | Σιδερίτης  (Siderìtis)  **(3, 4, 7, 8)** | AeP (e/o) | 0.31 | Essential oil and tea for infections of the gastrointestinal, urinary tract (antibacterial and antifungal action) and as antispasmodic for the stomach | Bahmani et al., 2015; Tunalier et al., 2004 |
| Lamiaceae | *Thymbra capitata* (L.) Cav.  **(AXL072)** | Θυμάρι  (Thimàri)  **(1, 3, 4, 5, 7, 8, 9)** | Fl (e/o), AeP, Lf | 0.18 | Tea from fresh and dry leaves for cough, constipation. Boiled leaves vapours for common colds. Fresh leaves for teeth pain. Decoction is used for duodenal ulcer (0,5-1,5mg of dry plant) |  |
| Lamiaceae | *Thymus sipyleus* Boiss.  **(AXL073)** | Θύμος  (Thìmos)  **(4, 7)** | AeP | 0.10 | Fresh or dry leaves in boiled water as expectorant, laxative. Vapours for pulmonary diseases and common colds. Tea (0,5-1,5mg) for duodenal ulcers |  |
| Lamiaceae | *Ziziphora taurica* subsp. *cleonioides* (Boiss.) P.H. Davis **(AXL004)** | Μέντα  (Mènta)  **(3)** | AeP (e/o) | 0.26 | Decoction for stomach pain and duodenal ulcer. The essential oil for external wounds | Meral et al., 2002 |
| Lauraceae | *Laurus nobilis* L.  **(AXL074)** | Δάφνη / Βάγια  (Dàfni)  **(3, 4, 7, 8)** | Lf, Fr, Fl (e/o) | 0.43 | Leaves as antiseptics, diuretics and emetogens. Oil from leaves has antibacterial and antimycotic action. Essential oil as anti-inflammatory | Dadalioglu et al., 2004; Sayyah et al., 2003 |
| Leguminosae | *Anthyllis hermanniae* L.  **(NEK001)** | Ανθυλλίς η ερμάννειος (Anthillìs)  **(1, 2, 3, 4, 5, 6, 7, 8, 9)** | Rt | 0.01 | Roots powder for kidney disease (1 teaspoon in 1 lt of water) | E.M.A^*^ |
| Leguminosae | *Glycyrrhiza glabra* Torr.  **(PROK007)** | Γλυκόρριζα  (Glikòriza)  **(3, 7)** | Rt | 0.17 | Powder of roots as spasmolytic (decoction). The black extract as expectorant (15-60g in 1 lt of water) | Wittschier et al., 2009 |
| Leguminosae | *Spartium junceum* L.  **(AXL075)** | Σπάρτο το βρουλόμορφο (Spàrto)  **(1, 2, 4, 6, 7, 8)** | Sh, Sd, Bk | 0.05 | Decoction of fresh barks have cardiotonic, diuretic action. Infusion of seeds and shouts for gastrointestinal diseases |  |
| Liliaceae | *Lilium candidum* L.  **(AXL076)** | Κρίνος  (Krìnos)  **(3, 4)** | Bu | 0.13 | Emollient cataplasm for ulcers. As a poultice on skin burns |  |
| Malvaceae | *Abutilon theophrastii* Medik.  **(AXL077)** | Αβούτιλο  (Avùtilo)  **(3,4)** | Lf, Rt | 0.01 | Tea from leaves and dry root for dysentery and fever. Poultice of leaves for ulcers |  |
| Malvaceae | *Malva sylvestris* L.  **(AXL078)** | Μολόχα  (Molòcha)  **(1, 2, 4, 5, 7, 8, 9)** | AeP, Fl, Lf | 0.40 | Tea from flowers and leaves as expectorants and lung diseases (10g in 1000 parts of water). A decoction from leaves as mouthwash for aphthae | Gasparetto et al., 2011 |
| Moraceae | *Ficus carica* L.  **(AXL079)** | Συκιά  (Sikià)  **(1, 2, 4, 6, 7, 8, 9)** | Lf, Fr | 0.13 | Leaves in boiled water for hemorrhoids. Boiled fruits as poultice for gingival inflammation |  |
| Moraceae | *Morus alba* L.  **(NEK029)** | Σκαμιά  (Skamià)  **(3, 4, 7, 8)** | Lf and Bk | 0.02 | Leaves for common cold, eyes inflammation, nosebleeds. Barks are used as antirheumatics, diuretics and antihypertensive | Wang et al., 2013 |
| Myrtaceae | *Eucalyptus camaldulensis* Dehnh.  **(AXL080)** | Ευκάλυπτος ο ρυγχωτός (Eukàliptos)  **(3, 4, 7, 8)** | e/o | 0.12 | Essential oil from leaves is a strong antiseptic and is used for lung diseases | Akin et al., 2010 |
| Myrtaceae | *Myrtus communis* L.subsp. c*ommunis*  **(KL146)** | Μυρσινιά  (Mirsinià)  **(3, 4, 6, 7, 8)** | Lf, Fr, e/o | 0.10 | Boiled leaves as hemostatics (cataplasm). Infusion from leaves for urinary tract infections, bronchitis. Essential oil as antiacne, gingival inflammation, scab | Akin et al., 2010 |
| Oleaceae | *Olea europaea L. var. europaea*  **(AXL081)** | Ελιά  (Elià)  **(1, 3, 4, 5, 6, 7, 8, 9)** | Lf | 0.04 | Decoction as expectorant, rheumatisms, cardiovascular diseases, tonsillitis, antispasmodic, oedemas, menstrual problems, hemorrhoids |  |
| Paeoniaceae | *Paeonia mascula* (L.) Mill. subsp *mascula*  **(AXL026)** | Παιώνια  (Peònia)  **(3, 4, 7)** | Rt | 0.01 | Roots as antispasmodic (powder). Tea from dry petals is used for cough, hemorrhoids and varicose veins |  |
| Pinaceae | *Pinus brutia* Ten.  **(AXL084)** | Πεύκη  (Pèfki)  **(1,3,4,7,8,9)** | Lf, Rn | 0.06 | Leaves as hemostatics (cataplasm). Infusion from roots for urinary tract infections, bronchitis. Essential oil as antiacne, gingival inflammation, scab |  |
| Platanaceae | *Platanus orientalis* L.  **(LS008)** | Πλάτανος  (Plàtanos)  **(1, 2, 4, 5, 7, 8)** | Lf, Bk, Fr | 0.07 | Bark is boiled with vinegar for diarrhoea, dysentery, and teeth pain. Fruits and bark are boiled and used as diuretic (infusion) |  |
| Plumbaginaceae | *Plumpago europea* L.  **(AXL085)** | Πλουμπάγκο η ευρωπαϊκή (Plubàgo)  **(1, 2, 3, 4, 7)** | Lf, Rt | 0.02 | Poultice from leaves powder for beard and scalp alopecia. Herb is decoted in a saucer with sulphur and lime and the mixture is then applied on the affected part |  |
| Polygonaceae | *Polygonum aviculare* L. subsp. *aviculare*  **(SM014)** | Πολύγωνο ή πολύκομπο (Polìgono)  **(1, 2, 4, 7, 8)** | AeP, Fr | 0.01 | All plant as anthelmintic, cholagogic (decoction) and hemostatic (cataplasm). For bacterial dysentery (decoction). For the relief of symptoms of common cold, minor inflammations in the mouth and throat (decoction) | E.M.A^*^ |
| Polygonaceae | *Rumex crispus* L.  **(KL186)** | Λάπαθο  (Làpatho)  **(1, 3, 4, 7)** | Lf, Rt | 0.13 | Root as laxative, cholagogic and as poultice or powder for wounds, ulcers. Fruits as antidiarrhoics | Yildirim et al., 2001 |
| Ranunculaceae | *Anemone coronaria* L.  **(AXL086)** | Λαλές  (Lalès)  **(1,3,4,5,7,8)** | Fl | 0.14 | Fresh flowers for rheumatisms and pain (cataplasm) |  |
| Rosaceae | *Agrimonia eupatoria* L.  **(G002)** | Αγριμόνια η ευπατόριος (Agrimònia)  **(1, 3, 7)** | AeP, Rt | 0.19 | All plant as strong astringent (infusion) and used for skin diseases and external haemorrhoids (cataplasm). For liver diseases and jaundice (1 teaspoon of leaves in 1lt of water) | E.M.A^*^ |
| Rosaceae | *Crataegus monogyna* Jacq.  **(AXL086)** | Τρικοκκιά  (Trikokià)  **(1, 3, 4, 7, 8)** | Fr, Bk | 0.03 | Tea from flowers for circulatory problems (10g of flowers in 1 lt of water 2-3/die for 1 month) |  |
| Rosaceae | *Potentilla recta* L.  **(AXL010)** | Ποτεντίλλη η όρθια  (Potentìli)  **(3, 4)** | AeP, Rt | 0.01 | Roots as strong astringent (decoction) and externally for hemorrhages (cataplasm) | Tomczyk and Latté, 2009 |
| Rosaceae | *Rosa canina* L.  **(AXL087)** | Άγριο τριανταφυλλιά (Triantafilià)  **(1,2,3,4,7,8)** | Fr, Bk | 0.02 | Decoction is used as tea for kidney problems. Fruits are boiled and mashed to obtain poultice for wound healing. Decoction as tea for diabetes mellitus, for haemorrhoids. As aphrodisiac and tonic (2-3kg of fresh fruits are boiled and sieved through muslin to obtain paste and honey is added. The paste is ingested on an empty stomach | Lattanzio et al., 2011 |
| Rosaceae | *Rubus sanctus* Schreb.  **(Ln024)** | Βάτος  (Vàtos)  **(1, 3, 4, 7, 8)** | Lf, Rt, Fr | 0.05 | Bark of leaves as poultice for diarrhoea. Roots for haemorrhoids (decoction) and boiled fruits for cystitis | Süntar et al., 2011 |
| Rutaceae | *Ruta chalepensis* L.  **(AXL088)** | Aπήγανος  (Apìganos)  **(2, 3, 4, 5, 7, 8, 9)** | AeP, Lf | 0.01 | Leaves boiled and the poultice is used for internal ear pain |  |
| Salicaceae | *Populus alba* L.  **(AXL089)** | Λεύκη  (Lèfki)  **(1, 3, 4, 8, 9)** | Bk | 0.14 | Analgesic, Anti-inflammatory, Antiseptic, Diuretic. (Ash of the bark externally on the affected parts) |  |
| Salicaceae | *Salix alba* L.  **(AXL090)** | Ιτιά  (Itià)  **(1, 2, 3, 4, 7)** | Bk | 0.04 | Pain, Inflammation, Fever (decoction) | Shara and Stohs, 2015 |
| Scrophulariaceae | *Scrophularia canina* L.  **(DR028)** | Σκροφουλαρία  (Scrofulària)  **(1, 4, 5, 6, 7, 8)** | AeP, Lf, Bk, Rt | 0.06 | All plant boiled as poultice for wounds, breast stretch marks. 10-20g of leaves in 1 lt of water as a decoction as laxative |  |
| Scrophulariaceae | *Verbascum ikaricum* Murb.  **(IK091)** | Βερμπάσκο  (Verbàsko)  **(7, 8, 9)** | AeP | 0.02 | Flowers in oil for otitis of the internal or medium ear |  |
| Scrophulariaceae | *Verbascum mucronatum* Lam. | Καπτούρα, βοιδόγλωσσα – voidòglossa  (Kaptùra)  **(3)** | AeP, Lf, Fl | 0.01 | Aerial parts of plant have antibacterial, antiviral and antifungal action. 10-20g of leaves in 1 lt of water, filtered, for bronchitis, asthma, tuberculosis, as expectorant. Dried or fresh flowers are boiled in milk and applied externally for pruritic conditions |  |
| Solanaceae | *Datura stramonium* L.  **(A-LS 002)** | Διαβολόχορτο  (Diavolòhorto)  **(1,2,3,4,7,8)** | Lf | 0.01 | Externally to relieve pain as a poultice. The fruit juice against dandruff. Very toxic for internal usage (to be avoided) | Soni et al., 2012 |
| Solanaceae | *Hyoscyamus albus* L.  **(AXL091)** | Υοσκύαμος  (Iòskamos)  **(1, 2, 3, 4, 5, 7, 8)** | Lf, Sd | 0.04 | Leaves and fruits boiled are used as decoction for kidney pain, antispasmodic and analgesic. Very toxic for internal usage (to be avoided) |  |
| Solanaceae | *Mandragora officinarum* L. **(BO008)** | Μανδραγούδας (Mandragùdas)  **(6, 7, 9)** | Rt | 0.01 | Juice from the finely grated root was applied externally to relieve rheumatic pains. Very toxic for internal usage (to be avoided) |  |
| Tiliaceae | *Tilia platyphyllos* Scop.  **(F024)** | Φλαμουριά  (Flamurià)  **(3, 4, 7)** | Fl, Lf, Bk | 0.13 | Bark is used for rheumatisms and uric acid (40g of bark in 1lt of water and boiled till 3/4 of total volume) | Yayalaci et al., 2014 |
| Urticaceae | *Parietaria judaica* L.  **(AXL092)** | Κολητσίδα  (Κolitsìda)  **(1,2,3,4,5,7,8,9)** | AeP, Lf | 0.21 | All plant antitussive (decoction) and as poultice on burns and wounds. Fresh herbs are pounded in mortar to obtain juice and applied to the affected area with eczema (cataplasm) |  |
| Urticaceae | *Urtica dioica* L.  **(AXL093)** | Τσουκνίδα  (Tsuknìda)  **(3, 4)** | AeP, Sh, Lf, Rt | 0.24 | All plant as antiasthmatic, diuretic, hypoglycaemic as tea. Leaves are boiled in milk to prepare poultice and then applied to the affected area (rheumatic pain). The roots as tea (infusion) for allergenic itching on the skin and for benign prostatic hyperplasia. The whole plant mixed with *Thymus* sp. for diabetes mellitus(infusion) | E.M.A^*^ |
| Valerianaceae | *Valeriana dioscoridis* Sm. **(AXL020)** | Βαλεριάνα  (Valeriàna)  **(1, 3, 4, 7, 8)** | Rt | 0.04 | 2-4g of root powder boiled with water for headaches |  |
| Vitaceae | *Vitis vinifera* L. subsp. *sylvestris* (C.C. Gmel.)  **(KL076b)** | Αμπέλι  (Ampèli)  **(1,3,4)** | Fr, Sd | 0.31 | Pounded fruits (fresh or dried) are applied to abscess to promote suppuration. Pounded dry raisin is applied to bruises to relieve the pain (cataplasm) |  |
| Zygophyllaceae | *Tribulus terrestris* L.  **(AXL094)** | Τρίβολας  (Trìvolas)  **(1,2,3,4,7,8)** | Sd, Fl, Fr | 0.02 | Seeds boiled as poultice as anthelmintic, diuretics. Dry fruits for headaches and stomatitis |  |

^a^ Local name of the medicinal plants are given in Greek language

^b^ Numbers represent collection sites indicated in Figure 1

^c^ Parts used: Gr, grains; Bk, bark; Lf, leaves; AeP, aerial parts; Fl, flowers; Fr, fruits; Rn, resins; Bu, bulbs; Rt, roots; Sd, seeds; Sh, shoots; Tb, tuber; Cn, cones; An, anther.

*E.M. A = European Medicines Agency / Herbal medicines for human use

^*^Use Value: UV=U/N, where U is the number of citations per plant species, and N is the number of the informants

**Table S2.** Endemic medicinal plant species

| **Plant Family** | **Botanical name** | **Location** | **Voucher specimen** |
| --- | --- | --- | --- |
| Αlliaceae | *Allium candargyi* Karavok. & Tzanoud. | Lesvos | AXL095 |
| Αlliaceae | *Allium pilosum* Sm. | Agios Efstratios | AGE001 |
| Amaryllidaceae | *Galanthus ikariae* Baker | Ikaria | IK095 |
| Asteraceae | *Anthemis rosea* Sm*.* | Samos | SAM096 |
| Asteraceae | *Anthemis scopulorum* Rech.f. | Samos | SAM097 |
| Asteraceae | *Carthamus leucocaulos* Sm. | Ikaria, Fourni | IK096 |
| Asteraceae | *Centaurea acicularis* Sm*.* | Samos, Ikaria, Fourni | SAM098 |
| Asteraceae | *Centaurea raphanina* Sm. subsp. *mixta* (DC.) Runemark | Agios Efstratios, Ikaria | IK096 |
| Asteraceae | *Centaurea rechingeri* Phitos | Samos | SAM098 |
| Asteraceae | *Centaurea xylobasis* Rech.f. | Samos | SAM099 |
| Asteraceae | *Crepis fraasii* Sch.Bip. | Lesvos, Chios, Samos, Ikaria | AXL096 |

| Asteraceae | *Leontodon graecus* Boiss. & Heldr. | Chios | CHI095 |
| --- | --- | --- | --- |
| Asteraceae | *Onopordum majorii* Beauverd | Samos, Ikaria, Fourni | SAM100 |
| Asteraceae | *Ptilostemon gnaphaloides* (Cirillo) Soják subsp. *pseudofruticosus* (Pamp.) Greuter | Ikaria | IK097 |
| Asteraceae | *Taraxacum graecum* Dahlst. | Lesvos, Chios, Ikaria | AXL097 |
| Boraginaceae | *Symphytum davisii* Wickens subsp.  *icaricum* (Pawl.) Stearn | Ikaria | IK098 |
| Brassicaceae | *Erysimum hayekii* (Jav.& Rech.f.) Polatschek | Chios, Samos | AXL098 |
| Brassicaceae | *Erysimum rechingeri* Javorka | Lemnos | CHI096 |
| Brassicaceae | *Erysimum senoneri* (Reut)Wettst. subsp*. icaricum* Snogerup | Ikaria | LIM001 |
| Brassicaceae | *Iberis runemarkii* Greuter & Burdet | Ikaria | IK098 |
| Brassicaceae | *Rorippa icarica* Rech. F. | Ikaria | IK099 |
| Caryophyllaceae | *Arenaria guicciardii* Heldr. | Lesvos, Chios, Samos | IK100 |
| Caryophyllaceae | *Paronychia chionaea* Boiss. | Lesvos, Chios, Samos | AXL099 |
| Geraniaceae | *Erodium vetteri* Barbey & Major | Samos | AXL100 |

| Hyacinthaceae | *Muscari kerkis* Karlén | Samos | SAM101 |
| --- | --- | --- | --- |
| Iridaceae | *Crocus cartwrightianus* Herb. | Ikaria | SAM102 |
| Lamiaceae | *Satureja icarica* P.H. Davis | Ikaria | IK101 |
| Lamiaceae | *Thymus samius* Ronniger & Rech.f. | Samos | IK102 |
| Orchidaceae | *Dactylorhiza kalopissii* E.Nelson subsp*. pythagorae* (Gölz & H.R.Reinhard ) Kreutz | Samos (mt. Karvouni) | SAM103 |
| Paeoniaceae | *Paeonia mascula* (L.) Mill*.*subsp.  *icarica* Tzanoud. | Ikaria | SAM104 |
| Plumbaginaceae | *Acantholimon aegaeum* F.K. Mey | Chios, Samos | IK103 |
| Plumbaginaceae | *Armeria icarica* J.R. Edm. | Ikaria | SAM105 |
| Plumbaginaceae | *Limonium palmare* (Sm.) Rech.f. | Ikaria, Fourni | IK104 |
| Polygonaceae | *Polygonum icaricum* Rech.f. | Lemnos, Chios, Ikaria | IK105 |
| Rubiaceae | *Asperula icarica* Ehrend. & Schönb. Tem. | Ikaria | IK106 |
| Rubiaceae | *Asperula lilaciflora* Boiss*.* | Chios, Ikaria | IK107 |
| Rubiaceae | *Asperula nitida* Sm. subsp*. mytilinica* Ehrend*.* | Lesvos, Chios | CHI096 |

| Rubiaceae | *Asperula samia* Christod. &  T. Georgiadis | Samos | AX101 |
| --- | --- | --- | --- |
| Rubiaceae | *Galium conforme* Krendl | Chios | SAM106 |
| Rubiaceae | *Galium pastorale* Krendl | Samos (mt. Kerki) | CHI097 |
| Rubiaceae | *Galium samium* Krendl | Samos, Ikaria | SAM107 |
| Scrophulariaceae | *Verbascum ikaricum* Murb. | Ikaria | SAM108 |
| Scrophulariaceae | *Digitalis cariensis*Jaub & Spach subsp. *ikarica* (P.H. Davis) Strid | Ikaria | IK108 |
| Veronicaceae | *Cymbalaria microcalyx* (Boiss.) Wettst.subsp. *dodecanesi* Greuter | Ikaria | SAM109 |
| Veronicaceae | *Veronica sartoriana* Boiss  & Heldr. | Samos | IK109 |

**Table S3.** FIC value of category of ailments

|  |  |
| --- | --- |
| **Ailments** | **FIC^*^** |
| Cardiovascular diseases | 0,161 |
| Gastrointestinal diseases | 0,319 |
| Neurological diseases | 0,571 |
| Antimicrobial | 0,245 |
| Dermatological diseases | 0,217 |
| Analgesic / Anti-inflammatory | 0,434 |
| Urinary diseases | 0,214 |
| Respiratory diseases | 0,411 |
| Hormonal diseases | 0,722 |

^*^FIC = N_ur_ – N_t_ / N_ur_ – 1, where N_ur_ refers to the number of citations in each category and N_t_ to the number of plant species used, with a range value from 0 to 1. Higher FIC (closer to 1) value indicates that relatively small number of plants are used by a large number of informants.

**Table S4.** Geographical coordinates of the Islands

| **Island** | **Geographical coordinates** |
| --- | --- |
| *Lemnos* | 39° 54′ 10″ N, 25° 13′ 13″ E |
| *Lesvos* | 39° 10′ 58″ N, 26° 12′ 10″ E |
| *Agios Efstratios* | 39° 31′ 8″ N, 25° 0′ 27″ E |
| *Chios* | 38° 22′ 59″ N, 26° 2′ 40″ E |
| *Psara* | 38° 34′ 10″ N, 25° 35′ 5″ E |
| *Oinousses* | 38° 31′ 19″ N, 26° 13′ 52″ E |
| *Samos* | 37° 43′ 41″ N, 26° 49′ 10″ E |
| *Ikaria* | 37° 35′ 58″ N, 26° 10′ 0″ E |
| *Fournoi* | 37° 35′ 27″ N, 26° 30′ 8″ E |

**Figure S1.**


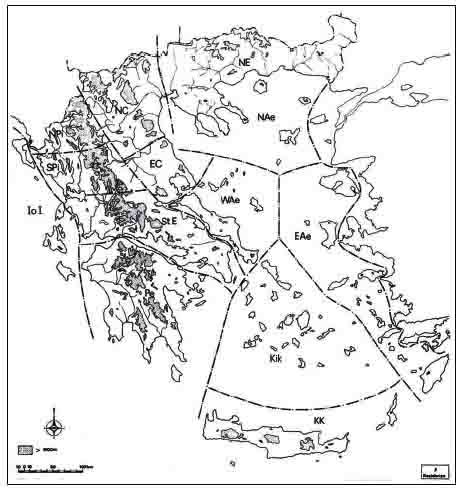


**Phytogeographical zones of Greece**

**Figure S2.**

**Total number of plant species recorded for every medicinal plant family**


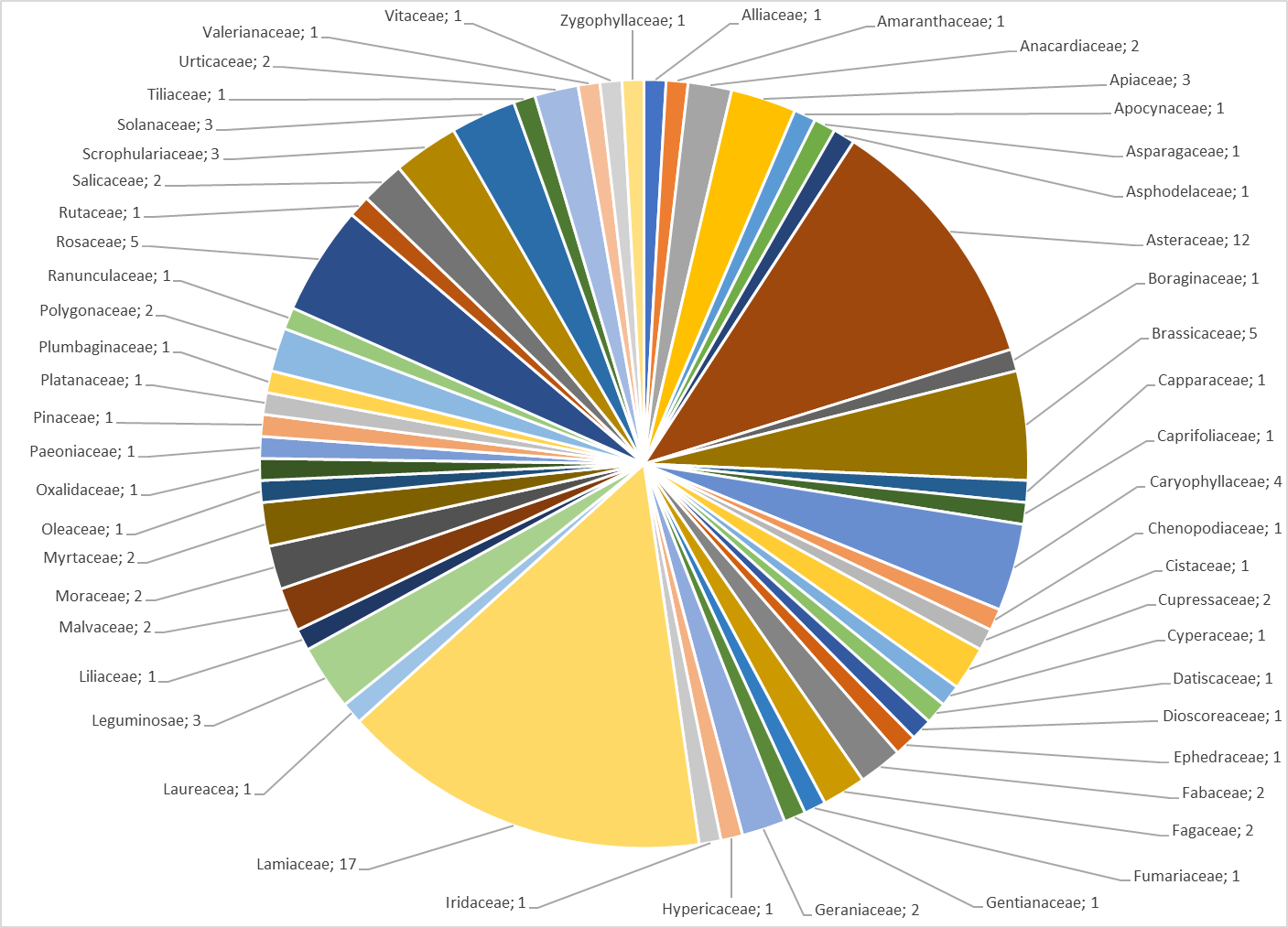

Supplement: Supplementary file 1 [file Table_1.doc]
